# An Innovative Collision-Free Image-Based Visual Servoing Method for Mobile Robot Navigation Based on the Path Planning in the Image Plan

**DOI:** 10.3390/s23249667

**Published:** 2023-12-07

**Authors:** Mohammed Albekairi, Hassen Mekki, Khaled Kaaniche, Amr Yousef

**Affiliations:** 1 Department of Electrical Engineering, College of Engineering, Jouf University, Sakakah 72388, Saudi Arabia; msalbekairi@ju.edu.sa; 2 NOCCS Laboratory, National School of Engineering of Sousse, University of Sousse, Sousse 4054, Tunisia; hassen.mekki@eniso.u-sousse.tn; 3 Electrical Engineering Department, College of Engineering, University of Business and Technology, Jeddah 21589, Saudi Arabia; a.yousef@ubt.edu.sa; 4 Engineering Mathematics Department, Faculty of Engineering, Alexandria University, Alexandria 5424041, Egypt

**Keywords:** mobile robot control, autonomous system, differential flatness, image-based visual servoing (IBVS), path planning, path tracking, collision-free, field of view (FOV)

## Abstract

In this article, we present an innovative approach to 2D visual servoing (IBVS), aiming to guide an object to its destination while avoiding collisions with obstacles and keeping the target within the camera’s field of view. A single monocular sensor’s sole visual data serves as the basis for our method. The fundamental idea is to manage and control the dynamics associated with any trajectory generated in the image plane. We show that the differential flatness of the system’s dynamics can be used to limit arbitrary paths based on the number of points on the object that need to be reached in the image plane. This creates a link between the current configuration and the desired configuration. The number of required points depends on the number of control inputs of the robot used and determines the dimension of the flat output of the system. For a two-wheeled mobile robot, for instance, the coordinates of a single point on the object in the image plane are sufficient, whereas, for a quadcopter with four rotating motors, the trajectory needs to be defined by the coordinates of two points in the image plane. By guaranteeing precise tracking of the chosen trajectory in the image plane, we ensure that problems of collision with obstacles and leaving the camera’s field of view are avoided. Our approach is based on the principle of the inverse problem, meaning that when any point on the object is selected in the image plane, it will not be occluded by obstacles or leave the camera’s field of view during movement. It is true that proposing any trajectory in the image plane can lead to non-intuitive movements (back and forth) in the Cartesian plane. In the case of backward motion, the robot may collide with obstacles as it navigates without direct vision. Therefore, it is essential to perform optimal trajectory planning that avoids backward movements. To assess the effectiveness of our method, our study focuses exclusively on the challenge of implementing the generated trajectory in the image plane within the specific context of a two-wheeled mobile robot. We use numerical simulations to illustrate the performance of the control strategy we have developed.

## 1. Introduction

In the context of 2D visual servoing, also known as image-based visual servoing (IBVS), one of the major challenges we face is obstacle avoidance. Our main objective is to use visual data from a 2D camera to control the movement of a robotic system, with the image the camera captures serving as our central reference point. The complexity of the task arises when we need to guide the robot to a specific destination while avoiding possible obstacles that may be in its path. This requires real-time analysis of visual data to detect the presence of obstacles, estimate their position and trajectory, and then adjust the robot’s path accordingly while ensuring that the visual target remains within the camera’s field of view (FOV). This task requires a combination of skills in computer vision, trajectory planning in the Cartesian plane, and control to ensure both environmental safety and mission accomplishment. In summary, obstacle avoidance in the context of 2D visual servoing is an essential and challenging task that requires an innovative and effective approach to ensure the success of robotic applications.

In many research studies, various approaches have been explored to solve this complex problem. For example, in [1], navigation is based on vanishing points to guide a quadcopter through corridors without colliding with walls. Other researchers, such as [2,3,4,5,6], have used different types of sensors, including stereo, monocular, and RGB-D cameras, to obtain three-dimensional information about the environment. They have also implemented techniques such as simultaneous localization and mapping (SLAM) to reconstruct maps of the environment, which are then used for motion planning and obstacle avoidance. Stereo cameras have also been used, as seen in the works of [7,8,9], combining visual, depth, and inertial information for navigation and obstacle avoidance. Studies like [10,11,12,13] have explored optical flow to find obstacles and estimate the speed difference between the robot and the obstacles. This is followed by trajectory planning and adaptive control to achieve navigation tasks.

In parallel, some studies have focused on the “teach and repeat” strategy, where a set of key images of the environment is captured, stored, and ordered for the robot’s navigation. This allows the robot to follow the same path multiple times, with examples such as [14,15,16,17,18,19]. A learning phase is required to capture the environment’s topology from these key images.

In a recent study, authors in [20] proposed a hierarchical visual servo control scheme for visual trajectory tracking of quadcopters in indoor environments. The control scheme is capable of handling three tasks, taking into account a hierarchy order: first, the collision avoidance task, then the visibility task, and finally, the visual task.

Path planning and tracking in the control loop can solve IBVS’s above issues and deal with complex scenes, including obstacles. Ideally, the idea is to generate a trajectory in the image plane and force the robot to follow it. According to [21], this is an extremely complex problem. To date, no control law generated, in a deterministic way, from a trajectory imposed in the image plane has been proposed. Despite this, there are many efforts being made. In [21], when the target is very far from the robot, the visual servoing algorithm may diverge. As a solution, the authors create a sequence of images between the image captured at the initial instant and the desired (final) image. They use a trajectory in 3D Cartesian space, which they project into the image plane. The trajectory is generated using the potential field method. Refs. [22,23] proposed a driving assistance system in which a trajectory in the image plane is generated. The outcomes of these two projects are used in a remote operation. The works presented in [24,25,26,27,28,29] seek to find a feasible trajectory in Cartesian space (3D) while avoiding obstacles and guaranteeing the visibility of the object in the image plane. An estimation of the pose of the robot and a priori knowledge of the obstacles are necessary. Once the trajectory is generated, an IBVS control algorithm called the Image-Based Tracker algorithm is used to ensure this trajectory. In [30], a 2D visual servoing method without camera calibration based on homographic projection is proposed. This method uses another form of the interaction matrix based on the homographic projection on a horizontal plane. The control law resembles that of classical visual servoing by replacing the interaction matrix with the proposed matrix. Since the proposed method does not allow direct control of the robot’s motion, leading to undesirable movements, Ref. [30] suggested adding a trajectory optimization method to the homographic projection. The idea is to generate a feasible trajectory in the projective homographic space. Subsequently, the large initial error is divided into small errors used to follow the discretized trajectory. The optimization of the trajectory is performed on the image plane. Ref. [31] attempted to determine an optimal trajectory using dynamic programming. The possible trajectories are divided into several smaller trajectories, and a cost function is integrated to penalize undesirable sub-trajectories. Constraints are added on the borders of the image (to guarantee the visibility of the scene), on the limits of the control, and on the limits of the joints of the robot. In this work, the authors did not consider the presence of obstacles. For this, a priori knowledge of the location of the obstacles in the Cartesian coordinate system is necessary. A navigation algorithm using visual memory is proposed in [32]. A keyframe topology map is generated from the movement of a camera over a model of the real scene. During navigation, a sequential comparison of stored images with captured images is performed. The desired trajectory here is defined by a number of waypoints. Each waypoint takes the form of a desired intermediate image. No direct planning on the image plane is performed.

In this paper, we propose a new method of path planning and tracking in the image plane in order to perform a visual servoing of a differential mobile robot using the concept of differential flatness. For the first time in the field of visual servoing based on path planning, it would be possible to generate any trajectory in the image plane and ensure its tracking while guaranteeing the controllability of the robot as well as the mechanical feasibility, high tracking accuracy, and position, velocity, and acceleration control on any desired trajectory and consequently on the robot.

We show that using the differential flatness of the system’s dynamics lets us limit arbitrary trajectories based on the number of points the object needs to reach in the image plane, creating a link between the current configuration and the desired configuration. The number of required points depends on the number of control inputs of the robot being used and determines the dimension of the flat output of the system. For a two-wheeled mobile robot, for instance, the coordinates of a single point on the object in the image plane are sufficient, whereas, for a quadrotor with four rotating motors, the trajectory needs to be defined by the coordinates of two points in the image plane. By ensuring precise tracking of the chosen trajectory in the image plane, we ensure that collision problems with obstacles and leaving the camera’s field of view are avoided. Our approach is based on the inverse problem principle, meaning that when any point on the object is selected in the image plane, it will in no way be occluded by obstacles or exit the camera’s field of view during movement. It is true that proposing any trajectory in the image plane can result in non-intuitive movements (back and forth) in the Cartesian plane. In the case of backward movement, the robot may collide with obstacles as it navigates without direct vision. Therefore, it is essential to perform optimal path planning that avoids backward movements. To evaluate the effectiveness of our method, we will focus on the specific case of a two-wheeled mobile robot. Numerical simulations are presented to demonstrate the efficiency of the proposed control strategy.

The following points outline the major contributions of this work:

▪ The proposed trajectory planning algorithm includes, implicitly, a permanent guarantee of target visibility and thus the impossibility of encountering an obstacle while ensuring robot controllability.
▪ The problem of a distant target no longer arises. The FOV is guaranteed even in the presence of obstacles using only a single monocular sensor.
▪ The integration of multiple sensors may require complex data fusion algorithms to combine information from different sources. With a single sensor, software complexity is reduced, which can simplify system development and maintenance.
▪ In the case of a mobile robot, the trajectory of a single point on the target object is sufficient. Any curve in the image plane between the two boundary points (initial and desired) can be ensured.
▪ Using a single point on the object as a descriptor provides greater robustness to the detection and recognition phases.
▪ Since this trajectory uses a certain number of points (called collocation points), we can introduce the concept of time between the points. This implies that we have just imposed a dynamic on this trajectory (in position and speed) and thus a dynamic on the robot.
▪ The imposed trajectory is physically feasible thanks to the concept of differential flatness (there is equivalence between differential flatness and controllability).
▪ No a priori knowledge of the 3D environment is necessary.
▪ The proposed algorithm is able to ensure robust tracking of the imposed trajectory.
▪ The time required to ensure that this trajectory can be fixed (within the limits of the robot’s physical constraints).

The paper’s structure is as follows: Section 2 exposes the problem formulation. In Section 3, we present the general control law design, including the flatness model, path planning, and path tracking process. The synthesis of the mobile robot control law is detailed in Section 4. Finally, Section 5 discusses simulation results that prove the effectiveness of the proposed method.

## 2. Problem Formulation

### 2.1. Differential Mobile Robot

**Figure 1:**
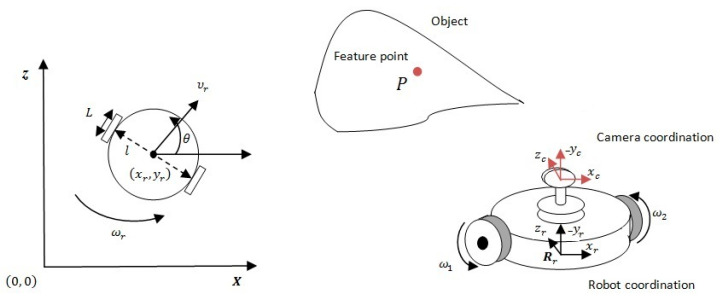
A two-wheeled mobile robot.

The mobile robot considered in this work is of the single-cycle type shown schematically in Figure 1. This robot is equipped with two independently controlled drive wheels and a freewheel, ensuring its stability.

$\left(x_{r},y_{r}\right)$ are midpoint coordinates between the two driving wheels, θ the orientation of the robot, $\omega_{1}$ and $\omega_{2}$ are velocities of the two driving wheels, l is the distance between the two driving wheels, and L is the diameter of a drive wheel. It is assumed that the robot movement occurs without sliding. The robot kinematic model can be expressed as follows:

(1) $$\left\{\begin{array}{c} {\dot{x}}_{r}=\upsilon_{r}cos\theta \\ {\dot{y}}_{r}=\upsilon_{r}sin\theta \\ \dot{\theta }=\omega_{r} \end{array}\right.$$

 $\upsilon_{r}$ is the linear speed of the robot given by

(2) $$\upsilon_{r}=\frac{L}{4}\left(\omega_{1}+\omega_{2}\right),$$

and $\omega_{r}$ is the angular velocity of the robot expressed by

(3) $$\omega_{r}=\frac{L}{2l}\left(\omega_{2}-\omega_{1}\right).$$

The mobile robot state is given by $q={\left[x_{r},y_{r},\theta \right]}^{T}$, and the two control inputs are $\upsilon_{r}$ and $\omega_{r}$.

### 2.2. Relations between Coordinate Systems

According to Figure 1, the robot is equipped with a camera. By utilizing the following homogeneous transformation matrix, we can calculate the robot–camera coordinate system transformation:

(4) $$T_{c}^{r}=\left[\begin{array}{cc} R_{c}^{r} & t_{c}^{r}\\ 0 & 1 \end{array}\right],$$

where $T_{c}^{r}\in R^{4\times 4}$. $R_{c}^{r}=\left(\begin{array}{ccc} r_{11} & r_{12} & r_{13}\\ r_{21} & r_{22} & r_{23}\\ r_{31} & r_{32} & r_{33} \end{array}\right)\in R^{3\times 3}$ is the rotation matrix. 

$t_{c}^{r}={\left[\begin{array}{ccc} t_{x} & t_{y} & t_{z} \end{array}\right]}^{T}\in R^{3}$ is the position vector where $t_{x},\;t_{y},\;t_{z}$ are the relative displacements between the robot coordinate system and the camera coordinate system. Since the camera is fixed on the base of the mobile robot, the robot–camera velocity relationship can be expressed as follows:

(5) $$\zeta_{r}^{r}={\left[0,\;0,\upsilon_{r},0,\omega_{r},0\;\right]}^{T}=\left[\begin{array}{cc} R_{c}^{r} & {s(t}_{c}^{r})R_{c}^{r}\\ 0_{3\times 3} & R_{c}^{r} \end{array}\right]\zeta_{c}^{c}.$$

In Equation (5), $\zeta_{c}^{c}={\left[\upsilon_{x},\upsilon_{x},\upsilon_{x},\omega_{x},\omega_{y},\omega_{z}\right]}^{T}$ represents the velocity of the camera expressed in the camera coordinate system. $\zeta_{r}^{r}$ is the velocity of the robot expressed in the robot coordinate system. ${s(t}_{c}^{r})$ is the skew-symmetric matrix of the vector $t_{c}^{r}$. $\upsilon_{r}$ and $\omega_{r}$ are, respectively, the translation and rotation velocity of the robot.

By simplifying Equation (5), we obtain

(6) $$\left[\begin{array}{c} \upsilon_{r}\\ \omega_{r} \end{array}\right]=\left[\begin{array}{cc} r_{33} & r_{22}t_{x}-r_{12}t_{y}\\ 0 & r_{22} \end{array}\right]\left[\begin{array}{c} \upsilon_{c}\\ \omega_{c} \end{array}\right],$$

where ${\left[\begin{array}{cc} \upsilon_{c} & \omega_{c} \end{array}\right]}^{T}$ is the camera velocity vector expressed in the camera coordinate system. The rotational velocities of the two drive wheels are given by the following expression:

(7) $$\left[\begin{array}{c} \omega_{1}\\ \omega_{2} \end{array}\right]=\left[\begin{array}{cc} L/2 & L/l\\ L/2 & -L/l \end{array}\right]\left[\begin{array}{c} \upsilon_{r}\\ \omega_{r} \end{array}\right]$$

### 2.3. Perspective Transformation Model

**Figure 2:**
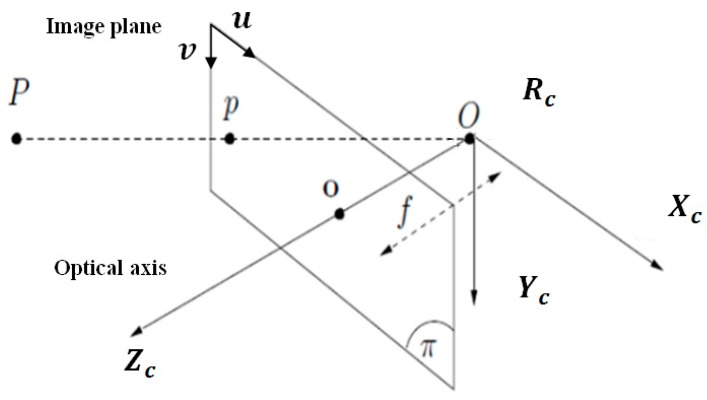
Perspective projection model.

Figure 2 shows the perspective transformation model.

Let P be any point in space. p represents the projection of P in the 2D plane called π. O is the center of projection. o is the intersection between the optical axis passing through O and the image plane. f is the focal length of the camera. $R_{c}$ is the camera coordinate system. Let $X=\left(X_{c},Y_{c},\;Z_{c}\right)$ the coordinates of the point P in the 3D Cartesian coordinate system; the projection of this point on the plane π is located at p whose coordinates are (x,y) in the plane π expressed in mm. The expressions of these coordinates are given by the following relations:

(8) $$\left\{\begin{array}{c} x=\frac{X_{c}}{Z_{c}}=\frac{\left(u-c_{u}\right)}{f.\alpha_{u}}\\ y=\frac{Y_{c}}{Z_{c}}=\frac{\left(v-c_{v}\right)}{f.\alpha_{v}} \end{array}\right..$$

The pair (u,v) represents the image point p coordinates expressed in pixels. $a=\left(c_{u},\;c_{v},\;f,\;\alpha_{u},\;\alpha_{v}\right)$ represents the camera intrinsic parameter: $\left(c_{u},\;c_{v}\right)$ are the coordinates of the principal point, f being the focal distance, and $\left(\alpha_{u},\;\alpha_{v}\right)$ are the vertical and horizontal scale factors expressed in pixel/mm. The time derivative of Equations (8) returns

(9) $$\left\{\begin{array}{c} \dot{x}=\dot{X_{c}}/Z_{c}-X_{c}\dot{Z_{c}}/{Z_{c}}^{2}=(\dot{X_{c}}-x\dot{Z_{c}})/Z_{c}\\ \dot{y}=\dot{Y_{c}}/Z_{c}-Y_{c}\dot{Z_{c}}/{Z_{c}}^{2}=(\dot{Y_{c}}-y\dot{Z_{c}})/Z_{c} \end{array}\right..$$

Using Equation (9), we can connect the velocity of the 3D point P to the velocity of the camera using Equation (9), which give

(10) $$\dot{X}=-v_{c}-\omega_{c}\;\cdot X.$$

Equation (10) can be expanded to obtain the following form:

(11) $$\left\{\begin{array}{c} \dot{X_{c}}=-v_{x}-\omega_{y}Z_{c}+\omega_{z}Y_{c}\\ {\dot{Y}}_{c}=-v_{y}-\omega_{z}X_{c}+\omega_{x}Z_{c}\\ \dot{Z_{c}}=-v_{z}-\omega_{x}Y_{c}+\omega_{y}X_{c} \end{array}\right.\;.$$

Using Equations (9) and (11) we can write

(12) $$\left\{\begin{array}{c} \dot{x}=-\frac{v_{x}}{Z_{c}}+\frac{xv_{z}}{Z_{c}}+xy\omega_{x}-\left(1+x^{2}\right)\omega_{y}+y\omega_{z}\\ \dot{y}=-\frac{v_{y}}{Z_{c}}+\frac{yv_{z}}{Z_{c}}+\left(1+y^{2}\right)\omega_{x}-xy\omega_{y}-x\omega_{z} \end{array}\right..$$

We can deduce the following compact form:

(13) $$\dot{X}=L_{s}\cdot \;V,$$

where $L_{s}$ is the interaction matrix, commonly known as the image’s Jacobian:

(14) $$L_{s}=\left[\begin{array}{ccc} -\frac{1}{Z_{c}} & 0 & \frac{x}{Z_{c}}\\ 0 & -\frac{1}{Z_{c}} & \frac{y}{Z_{c}} \end{array}\begin{array}{ccc} \;\;\;\;\;xy & -\left(1+x^{2}\right) & y\\ \;\;\;\;\;\;1+y^{2} & -xy & -x \end{array}\right],$$

$L_{s}$ ensures the link between the variation in the position of the point p and, consequently, the variation of its coordinates in pixels (u, v) and the velocity of the camera $V={\left[\begin{array}{ccc} v_{x} & v_{y} & v_{z} \end{array}\;\begin{array}{ccc} \omega_{x} & \omega_{y} & \omega_{z} \end{array}\right]}^{T}$. In the interaction matrix expression, $Z_{c}$ represents the depth of the point P. Any control law using this form of interaction matrix must first estimate the value of $Z_{c}$. Intrinsic parameters of the camera are involved in the calculation of x and y. Since the movement of the robot is performed in the 2D plane, the linear and angular velocities are in the direction of the $Z_{c}$ axis and the $Y_{c}$ axis, respectively, the interaction matrix can be reformulated as follows:

(15) $$L_{s}=\left[\begin{array}{cc} \frac{x}{Z_{c}} & -\left(1+x^{2}\right)\\ \frac{y}{Z_{c}} & -xy \end{array}\right].$$

## 3. Proposed Control Law Design

### 3.1. Flatness of a Model

A system is described by the equation

(16) Φ(X ˙(t),X(t),u(t))=0,

where X(t) denotes the state and u(t) denotes the command, is flat if there is a vector z(t) such that

(17) $$z(t)=h(X\left(t\right),\;u\left(t\right),\;u^{\left(1\right)}\left(t\right),\;\ldots ,\;u^{\left(\delta \right)}),$$

whose components are differentially independent and have two functions A(.) and B(.) such that

(18) $$X(t)=A(z\left(t\right),\;z^{\left(1\right)}\left(t\right),\;\ldots ,\;z^{\left(\alpha \right)}\left(t\right)),$$

(19) $$u\left(t\right)=B\left(z\left(t\right),\;z^{\left(1\right)}\left(t\right),\;\ldots ,\;z^{\left(\beta \right)}\left(t\right)\right),$$

where α,β, and δ are three finite integers. This formulation refers to the vector z(t) as the flat output of the system. By introducing the functions A(.) and B(.), this flat output is composed of a set of variables that enables parameterization of all other system variables: the state, the command, and also the output y (t). Indeed, if the output of the system is defined by a relation of the form $y(t)=\Psi (X\left(t\right),\;u\left(t\right),\;\ldots ,\;u^{\left(p\right)}\left(t\right))$ then necessarily the quantities described in (18) and (19) make it possible to affirm that there exists an integer γ such that

(20) $$y(t)=C(z\left(t\right),\;\ldots ,\;z^{\left(\gamma \right)}\left(t\right)).$$

The flat output groups all of the free (unconstrained) variables of the system since the components of z(t) are differentially independent. However, we can also consider, on the basis of Equation (17), that the flat output z(t) only depends on the state and the command. This would make it an endogenous variable of the system, in contrast to the state of an observer, which would be an example of an exogenous variable of the observed system. In addition, Lie-Bäcklund’s notion of differential equivalence [33] shows that the number of components of z(t) is the same as the number of components of the command:

(21) dim⁡z(t)=dim⁡u(t).

This is a basic property that permits one to know how many free variables must be found on a model to show that it is flat. One of the advantages of the flatness property is that the previous definition is not restricted to state models but to any model of the form

(22) $$\Phi (X^{\left(n\right)}\left(t\right),\;\ldots ,\;X^{\left(1\right)}\left(t\right),\;X\left(t\right),\;u^{\left(m\right)}\left(t\right),\;\ldots ,\;u^{\left(1\right)},\;u\left(t\right))=0.$$

This means that we can start right from the equations that describe how the system works. We do not have to rewrite all the equations into an equation of state. 

### 3.2. Path Planning in the Image Plane 

From Equation (19), if we decide to obtain, for the flat system described by Equation (16), the trajectory: $z_{d}(t)$ for t from $t_{0}$ to $t_{f}$, it is sufficient to apply, on the same time segment, the open loop control given is

(23) $$u_{d}(t)=B(z_{d}\left(t\right),\;z_{d}^{\left(1\right)}\left(t\right),\;\ldots ,\;z_{d}^{\left(\beta \right)}\left(t\right)).$$

Assuming a perfect model, we will then have, for t from $t_{0}$ to $t_{f}$, $z(t)=z_{d}\;(t)$, and therefore

(24) $$X\left(t\right)=X_{d}\left(t\right)=A\left(z_{d}\left(t\right),\;z_{d}^{\left(1\right)}\left(t\right),\;\ldots ,\;z_{d}^{\left(\alpha \right)}\left(t\right)\right),$$

(25) $$y(t)=y_{d}(t)=C(z_{d}\left(t\right),\;z_{d}^{\left(1\right)}\left(t\right),\;\ldots ,\;z_{d}^{\left(\gamma \right)}\left(t\right)).$$

The only constraint is that the desired trajectory on the flat output must necessarily be at least $max\left(\alpha ,\beta ,\gamma \right)$ times differentiable on $\left[t_{0}\;t_{f}\right]$. To ensure the differentiability constraint throughout the entire trajectory, we generally envisage, and without being restrictive, piecewise polynomial trajectories, interpolation polynomials, or $C^{\infty }$ functions with, most of the time, continuity conditions at the start and at the arrival. We can also impose passing or reversal points or also avoidance trajectories planned according to possible events.

### 3.3. Asymptotic Trajectory Pursuit 

With knowledge of Equation (19), the following command is proposed:

(26) $$u(t)=B(z\left(t\right),\;z^{\left(1\right)}\left(t\right),\;\ldots ,\;z^{\left(\beta -1\right)}\left(t\right),\;\vartheta (t)),$$

where ϑ(t) is a new command. When $\frac{\partial B(.)}{\partial z^{\left(\beta \right)}}$ is locally invertible, this leads to the following decoupled system:

(27) $$z^{\left(\beta \right)}(t)=\vartheta (t).$$

This result is to be compared to the linearization and to the decoupling by looping of the nonlinear systems, which are always conditioned by the stability of the zeros of the system [34]. Indeed, we obtain here an unconditional decoupling and linearization (note that this property is at the origin of the choice of the term flatness). However, it is obvious that an additional stabilization loop is necessary; ϑ(t) then becomes

(28) $$\vartheta (t)=z_{d}^{\left(\beta \right)}(t)+\sum_{i=0}^{\beta -1}k_{i}(z_{d}^{\left(i\right)}\left(t\right)-z^{\left(i\right)}\left(t\right)).$$

Let $K(p)=p^{\beta }+\sum_{i=0}^{\beta -1}k_{i}p^{i}$ is a diagonal matrix whose elements are polynomials with negative real part roots, u(t) then becomes

(29) $$u=B\left(z,\;\ldots ,\;z^{\left(\beta -1\right)},\;z_{d}^{\left(\beta \right)}(t)+\sum_{i=0}^{\beta -1}k_{i}(z_{d}^{\left(i\right)}\left(t\right)-z^{\left(i\right)}\left(t\right))\right),$$

or

(30) $$u=\Phi (z,\;\ldots ,\;z^{\left(\beta -1\right)},K\left(p\right)z_{d}\left(t\right)),$$

which makes it possible to ensure an asymptotic trajectory pursuit with

(31) $$\underset{t\to \infty }{\lim}\left(z_{d}\left(t\right)-z(t)\right)=0.$$

As z(t) and all its derivatives are endogenous variables of the process, the looping u(t) is called an endogenous looping.

## 4. Synthesis of the Proposed Mobile Robot Control Law

We showed in [35] that the coordinates of a single point of the object in the image plane p(x,y), can act as a flat output for the mobile robot. To simplify the problem, we can consider, in Figure 1, the coordinate system of the camera confused with the coordinate system of the robot. In this case, we can write

(32) $$\left(\begin{array}{c} \dot{x}\\ \dot{y} \end{array}\right)=\left(\begin{array}{cc} \frac{x}{Z_{c}} & -\left(1+x^{2}\right)\\ \frac{y}{Z_{c}} & -xy \end{array}\right)\left(\begin{array}{c} v_{r}\\ \omega_{r} \end{array}\right).$$

The two mobile robot control inputs are given by

(33) $$\left(\begin{array}{c} v_{r}\\ \omega_{r} \end{array}\right)={\left(\begin{array}{cc} \frac{x}{Z_{c}} & -\left(1+x^{2}\right)\\ \frac{y}{Z_{c}} & -xy \end{array}\right)}^{-1}\left(\begin{array}{c} \dot{x}\\ \dot{y} \end{array}\right).$$

By explaining the inverse of the interaction matrix, we obtain the following:

(34) $$\left(\begin{array}{c} v_{r}\\ \omega_{r} \end{array}\right)=\left(\begin{array}{cc} -\frac{x}{Z_{c}} & \frac{Z_{c}\left(1+x^{2}\right)}{y}\\ -1 & \frac{x}{y} \end{array}\right)\left(\begin{array}{c} \dot{x}\\ \dot{y} \end{array}\right).$$

Utilizing the concept of differential flatness provided by Hagenmeyer-Delaleau in [36], we can achieve a precise linearization. The resultant linearized system is comparable to a system with the following integration form:

(35) $$\left\{\begin{array}{c} \dot{x}=\vartheta_{x}\\ \dot{y}=\vartheta_{y} \end{array}\right.,$$

where $\vartheta_{x}$ and $\vartheta_{y}$ are the two auxiliary control inputs to be specified, given by Equation (28), which enable the asymptotic pursuit of the planned trajectory. The final form of the control law for the mobile robot is as follows:

(36) $$\left(\begin{array}{c} v_{r}\\ \omega_{r} \end{array}\right)=\left(\begin{array}{cc} -\frac{x}{Z_{c}} & \frac{Z_{c}\left(1+x^{2}\right)}{y}\\ -1 & \frac{x}{y} \end{array}\right)\left(\begin{array}{c} \vartheta_{x}\\ \vartheta_{y} \end{array}\right).$$

The two auxiliary control inputs ensuring the asymptotic tracking of the desired trajectory are given by

(37) $$\vartheta_{x}={\dot{x}}^{*}+k_{1}\left(x^{*}-x\right),$$

(38) $$\vartheta_{y}={\dot{y}}^{*}+k_{2}\left(y^{*}-y\right).$$

Let us consider $e_{x}=x^{*}-x$ and $e_{y}=y^{*}-y$. The error dynamics can be written as follows:

(39) $${\dot{e}}_{x}+k_{1}e_{x}=0,$$

(40) $${\dot{e}}_{y}+k_{2}e_{y}=0,$$

where $k_{1}$ and $k_{2}$ are chosen so that the error dynamics are asymptotically stable. In this case, it suffices to take $k_{1}>0$ and $k_{2}>0,$ which ensures an asymptotic pursuit of the desired trajectory $\left(x^{*},\;y^{*}\right)$. 

**Figure 3:**
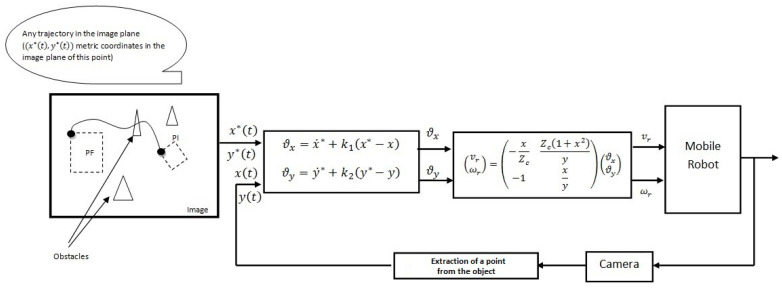
Proposed control scheme.

Figure 3 shows the proposed control scheme.

## 5. Simulation Results

**Figure 4:**
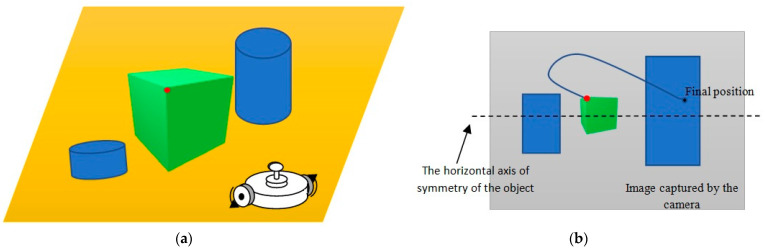
Simulation environment. (**a**) Three-dimensional environment; (**b**) image captured using the camera (size 1024 × 1024). Red point represents a single point (detected) in the object.

To demonstrate the efficiency of our control algorithm, we propose an arbitrary trajectory in the image plane which connects the initial and final position of any point or P of the object, except those that are situated along the horizontal axis of symmetry of the object. Indeed, the movement of the mobile robot is conducted in the 2D plane (presented in yellow in Figure 4); the horizontal axis of symmetry of the object remains invariant during the movement of the robot. Consequently, the dynamics of any point situated along the horizontal axis always remain zero or constant (unless the object is centered in the image). This situation contradicts the principle of differential flatness, which asserts that knowledge of the dynamics of the flat output allows for deducing the dynamics of all variables in the system. If the dynamics along the y-axis remain zero or constant, it creates a singularity, also known as the controllability problem. It is important to emphasize that it is possible to select any trajectory connecting the starting point to the endpoint, even by freehand drawing on the screen and using an interpolation algorithm to generate an analytical expression for this trajectory. In our specific example, we will choose a polynomial trajectory due to its simplicity in calculating derivatives.

Since we can impose a dynamic (in velocity and acceleration) on any desired trajectory, we propose a polynomial-type trajectory. Let $P_{i}=\left(x_{i}^{*},\;y_{i}^{*}\right)$, the initial position, in pixels, of a point P of the object in the image plane at time $t_{i}$, and $P_{f}=\left(x_{f}^{*},\;y_{f}^{*}\right)$, its final position at time $t_{f}$. Let us suppose we want to have a trajectory connecting these two points pass through a maximum. Let us consider, for example, the coordinate point $\left(\frac{x_{f}^{*}+x_{i}^{*}}{2},\;2y_{f}^{*}-y_{i}^{*}\right),$ which represents the maximum of a curve between $y_{i}^{*}$ and $y_{f}^{*}$. It should be noted here that the initial situation and the desired final situation must be taken by the camera installed on the real robot. We propose the following dynamic given by a slow start, acceleration in the middle of the trajectory, and finally, a slow convergence. The desired trajectory $y^{*}\left(x^{*}\right)$ must therefore satisfy the following constraints:

(41) $$\begin{array}{c} y^{*}\left(x_{i}^{*}\right)=y_{i}^{*},\\ y^{*}\left(x_{f}^{*}\right)=y_{f}^{*},\\ y^{*}\left(\frac{x_{f}^{*}+x_{i}^{*}}{2}\right)=2y_{f}^{*}-y_{i}^{*},\\ \frac{dy^{*}}{dx^{*}}\left(\frac{x_{f}^{*}+x_{i}^{*}}{2}\right)=0,\\ \frac{d^{2}y^{*}}{d^{2}x^{*}}\left(\frac{x_{f}^{*}+x_{i}^{*}}{2}\right)<0. \end{array}$$

As an example, we suggest the following polynomial equation in $x^{*}$ that satisfies the conditions stated in (41):

(42) $$y^{*}\left(x^{*}\right)=y_{i}^{*}+\left(y_{f}^{*}-y_{i}^{*}\right)\left(\frac{x^{*}-x_{i}^{*}}{x_{f}^{*}-x_{i}^{*}}\right)\left(9-12\left(\frac{x^{*}-x_{i}^{*}}{x_{f}^{*}-x_{i}^{*}}\right)+4{\left(\frac{x^{*}-x_{i}^{*}}{x_{f}^{*}-x_{i}^{*}}\right)}^{2}\right).$$

Therefore, it is necessary to create a variation of $x^{*}\left(t\right)$, which satisfies the following boundary conditions:

(43) $$x^{*}\left(t_{i}\right)=x_{i}^{*},\;{\dot{x}}^{*}\left(t_{i}\right)=0,\;\cdots ,\;x^{*(5)}\left(t_{i}\right)=0,$$

(44) $$x^{*}\left(t_{f}\right)=x_{f}^{*},\;{\dot{x}}^{*}\left(t_{f}\right)=0,\;\cdots ,\;x^{*(5)}\left(t_{f}\right)=0.$$

This produces the following 11-degree polynomial:

(45) $$\begin{array}{c} x^{*}\left(t\right)=x_{i}^{*}+\left(x_{f}^{*}-x_{i}^{*}\right)\sigma^{6}\left(t\right)(462-1980\sigma \left(t\right)+3465\sigma^{2}\left(t\right)-3080\sigma^{3}\left(t\right)+\\ 1386\sigma^{4}\left(t\right)-252\sigma^{5}\left(t\right)\left(t\right)), \end{array}$$

with

(46) $$\sigma \left(t\right)=\frac{t-t_{i}}{t_{f}-t_{i}}.$$

**Figure 5:**
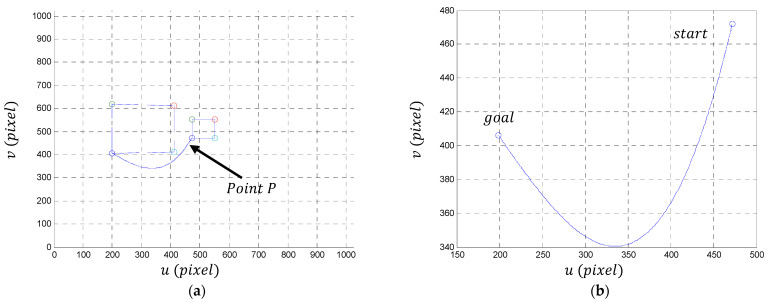

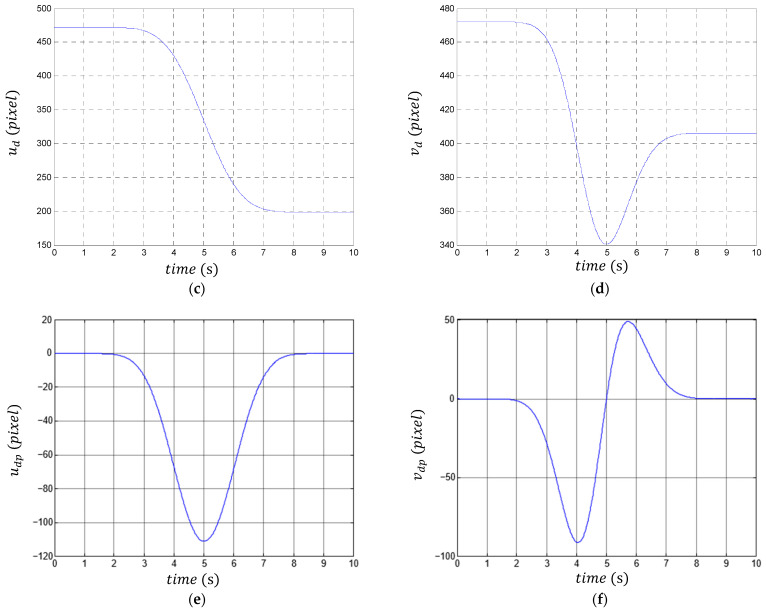
(**a**,**b**) The desired trajectory in the image plane of a point P of the object (The big and small boxes represent respectively the desired and initial target position—Only the bleu point is used to generate control laws); (**c**,**d**) the pixel variation in the desired trajectory; (**e**,**f**) the accelerations (in pixel) of the desired trajectory.

The dynamics of the desired trajectory are described in Figure 5. Figure 5a,b present the desired trajectory, in position, which connects the two points to the limits in the image plane and which passes through a maximum. Figure 5c,d show the velocity dynamics of this trajectory, and Figure 5e,f give the acceleration dynamics. We impose on the robot a slow start, acceleration in the middle of the trajectory, and finally, a slow convergence. Camera intrinsic parameters are considered as follows: $\left(c_{u},\;c_{v}\right)=(\frac{1024}{2},\frac{1024}{2})$; f=0.718 and $\left(\alpha_{u},\;\alpha_{v}\right)=(800,\;800)$.

### 5.1. Simulation Conditions

We consider an object described by four points. Figure 5a shows this rectangular-shaped object in its initial position (the small blue rectangle) and in the final position (the large blue rectangle). Our algorithm uses only one point (denoted P in Figure 5a) to generate the control law. The initial position $P_{i}$ and the desired position $P_{d}$ of the object in the image plane are given by the coordinates of the following characteristic points of the object (in blue the coordinates of point P):

(47) $$P_{i}=\left(\begin{array}{cc} 472 & 472\\ 472 & 552 \end{array}\;\;\;\;\begin{array}{cc} 552 & 552\\ 552 & 472 \end{array}\right),$$

(48) $$P_{d}=\left(\begin{array}{cc} 199 & 199\\ 406 & 618 \end{array}\;\;\;\;\begin{array}{cc} 411 & 411\\ 613 & 411 \end{array}\right).$$

The simulation parameters are chosen as follows: $L=0.3\;m,R=0.1\;m,k_{1}=k_{2}=100,$ T=10 s, and $Z_{c}=2.5\;m$.

### 5.2. Results and Interpretations

**Figure 6:**
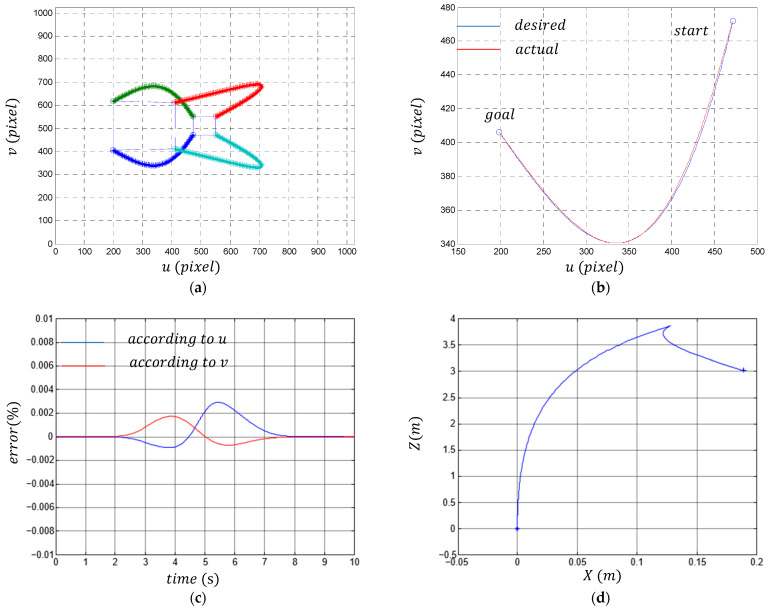

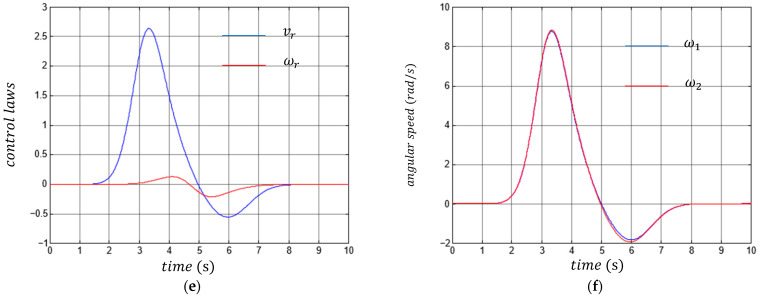
(**a**) The trajectory made by the four points (The big and small boxes represent, respectively, the desired and initial target positions; blue, green, red, and turquoise thick lines represent the four point trajectories in the image plane when the robot moves); (**b**) the actual and the desired trajectory in the image plane of a point P of the object; (**c**) the percentage of the error of the performed trajectory according to u and according to v; (**d**) the trajectory performed by the robot in the 2D Cartesian plane; (**e**) the evolution of the two commands $v_{r}$ and $\omega_{r}$ applied to the robot; (**f**) the variations in the two angular speeds $\omega_{1}$ and $\omega_{2}$ of the two driven wheels.

The simulation results are given in Figure 6. Figure 6a shows the trajectories performed by the four points. The desired end position and the reached end position of the four points are:

(49) $$P_{d}=\left(\begin{array}{cc} 199 & 199\\ 406 & 618 \end{array}\;\;\;\;\begin{array}{cc} 411 & 411\\ 613 & 411 \end{array}\right),$$

(50) $$P_{a}=\left(\begin{array}{cc} 199 & 199\\ 406 & 618 \end{array}\;\;\;\;\begin{array}{cc} 412 & 412\\ 612 & 410 \end{array}\right).$$

We notice that the two positions are almost similar; this proves that a single object point is enough to perform 2D visual servoing in the case of a mobile robot. Figure 6b represents the performed trajectory and the desired trajectory of a point P of the object in the image plane. We notice a high accuracy of the tracking process. The percentages of errors between the performed trajectory and the desired trajectory do not exceed 0.3% (Figure 6c). Figure 6d represents the movement performed by the robot in the Cartesian plane. We note that the robot has come and gone to reach the final position, which is normal since we have proposed any trajectory in the image plane, which necessarily generates any trajectory in the Cartesian plane. Figure 6e represents the two control laws $v_{r}$ and $\omega_{r}$ applied to the robot. Note that both controls are continuous and smooth. We also notice that the two control laws begin with a slow variation (a slow start of the robot), an acceleration in the middle, and a slow convergence. This proves that we have just controlled the dynamics of the robot via the choice of the desired trajectory in the image plane. The variation in the two rotational speeds of the two driving wheels is given in Figure 6f.

## 6. Conclusions

In this article, we introduced a novel approach to 2D visual servoing in complex environments containing obstacles. Our method only makes use of visual data obtained by a single monocular sensor. The fundamental idea is to manage and control the dynamics associated with any trajectory generated in the image plane. By ensuring precise tracking of the selected trajectory in the image plane, we prevent issues such as collisions with obstacles and loss of camera visibility. In the specific context of this work, which focuses on a mobile robot, we have demonstrated using the concept of differential flatness that the use of a single point on the object in the image plane as a descriptor is sufficient. In the future, this approach could be extended to robots with multiple degrees of freedom, such as manipulator arms or quadcopters, using other points on the object to represent the flat output dimension of the robot. It is important to note that proposing an arbitrary trajectory in the image plane can lead to non-intuitive movements, including back-and-forth motions, in the Cartesian plane. This can create a problem, especially when the robot is moving backward, which could result in collisions with obstacles as the robot moves without direct vision. Therefore, in future work, it would be essential to develop optimal trajectory planning that avoids backward movements. We were able to impose a dynamic in position, velocity, and acceleration on the robot by using the concept of flatness. It thus becomes possible to decide on any trajectory in the image plane of a primitive initially visible by the robot so that the robot executes the necessary movement that guarantees the exact following of this trajectory. In this paper, we were interested in visual servoing in the case of a static target. Since we have demonstrated that it is possible to control positions, velocities, and accelerations in the image plane, we can also use the proposed concept to carry out dynamic visual servoing.

## Data Availability

Data are contained within the article.
